# Developmental Origins of Kidney Disease: Why Oxidative Stress Matters?

**DOI:** 10.3390/antiox10010033

**Published:** 2020-12-30

**Authors:** Chien-Ning Hsu, You-Lin Tain

**Affiliations:** 1 Department of Pharmacy, Kaohsiung Chang Gung Memorial Hospital, Kaohsiung 833, Taiwan; cnhsu@cgmh.org.tw; 2 School of Pharmacy, Kaohsiung Medical University, Kaohsiung 807, Taiwan; 3 Department of Pediatrics, Kaohsiung Chang Gung Memorial Hospital and Chang Gung University College of Medicine, Kaohsiung 833, Taiwan; 4 Institute for Translational Research in Biomedicine, Kaohsiung Chang Gung Memorial Hospital and Chang Gung University College of Medicine, Kaohsiung 833, Taiwan

**Keywords:** antioxidant, hypertension, nitric oxide, asymmetric dimethylarginine, chronic kidney disease, oxidative stress, developmental origins of health and disease (DOHaD), nephrogenesis, reactive oxygen species

## Abstract

The “developmental origins of health and disease” theory indicates that many adult-onset diseases can originate in the earliest stages of life. The developing kidney has emerged as being particularly vulnerable to adverse in utero conditions leading to morphological and functional changes, namely renal programming. Emerging evidence indicates oxidative stress, an imbalance between reactive oxygen/nitrogen species (ROS/RNS) and antioxidant systems, plays a pathogenetic role in the developmental programming of kidney disease. Conversely, perinatal use of antioxidants has been implemented to reverse programming processes and prevent adult-onset diseases. We have termed this reprogramming. The focus of this review is twofold: (1) To summarize the current knowledge on oxidative stress implicated in renal programming and kidney disease of developmental origins; and (2) to provide an overview of reprogramming effects of perinatal antioxidant therapy on renal programming and how this may prevent adult-onset kidney disease. Although early-life oxidative stress is implicated in mediating renal programming and adverse offspring renal outcomes, and animal models provide promising results to allow perinatal antioxidants applied as potential reprogramming interventions, it is still awaiting clinical translation. This presents exciting new challenges and areas for future research.

## 1. Introduction

About 10% of the world’s population has chronic kidney disease (CKD) [1]. An estimated around 5–10 million people die per year from kidney disease globally [2]. Adult kidney disease can originate in early life [3,4] and; therefore, World Kidney Day 2016 informed the public about the need to focus on kidney disease in childhood and the antecedents [5]. The developing kidney is extraordinarily vulnerable to the effects of adverse environmental events, leading to functional and structural changes, namely renal programming [6]. The phenomenon of suboptimal conditions during organ development leading to increased risk of disease later in life is now termed “developmental origins of health and disease” (DOHaD) [7].

Clinical and experimental studies have provided support to the link between the DOHaD concept and kidney disease and demonstrated several mechanisms implicated [3,4,8,9,10]. Among them, oxidative stress plays a significant role in developmental origins of kidney disease [11,12]. Oxidative stress is defined as an imbalance between pro-oxidant molecules, in particular reactive oxygen species (ROS) and reactive nitrogen species (RNS), and antioxidant defenses, in favor of the first. ROS play a dual role in pregnancy [13,14]. Moderate ROS levels contribute to normal organogenesis and cell differentiation, while their overproduction adversely affects pregnancy and fetal outcomes. Additionally, overproduction of ROS resulting in oxidative stress reduces the bioavailability of nitric oxide (NO), a crucial mediator of maternal and fetal homeostasis in gestation [15]. Emerging evidence indicates the imbalance between ROS and NO has been implicated in developmental origins of kidney disease [16,17,18].

Since oxidative stress is considered as a key mechanism underlying CKD, it is logical to hypothesize that antioxidant therapy would be potential therapy to optimize renal outcomes. However, application of antioxidants clinically to patients with kidney disease has been met with limited success. Although antioxidant therapy provides renal benefits for patients with advanced CKD, it does not reduce the risk of cardiovascular disease or all-cause mortality [19,20].

The idea from DOHaD research opens up new avenues to reverse the programming process by early intervention aiming to prevent developmental origins of kidney disease in adulthood via so-called reprogramming [10]. Animal studies that have been published recently support that maternal antioxidant therapy can work as a reprogramming strategy to prevent hypertension programmed by diverse maternal insults [21]. Nevertheless, little is known on whether gestational supplementing with antioxidants can be protective on kidney disease programmed by a variety of early-life insults.

The aim of this review is to give an overview of oxidative stress implicated in kidney disease of developmental origins. The ROS-NO imbalance will be a special focus, and its programming effect on nephron endowment will be discussed. Finally, the use of antioxidants as a reprogramming approach to protect offspring against developmental origins of kidney disease will be summarized.

We retrieved related literature from all articles indexed in PubMed/MEDLINE. We used different combinations of keywords as follows: “antioxidants”, “chronic kidney disease”, “developmental programming”, “DOHaD”, “free radicals”, “offspring”, “mother”, “nephrogenesis”, “nephron”, “nitric oxide”, “oxidative stress”, “pregnancy”, “progeny”, “reprogramming”, “reactive oxygen species”, “reactive nitrogen species”, and “uremia”. Additional studies were then selected and evaluated based on references from eligible articles. The last search was conducted on 30 November 2020.

## 2. Oxidative Stress and Developmental Programming

### 2.1. ROS and NO Signal

ROS include free radicals such as superoxide anion (O_2_^−^) and hydroxyl anion (OH^−^) and non-radical molecules like hydrogen peroxide (H_2_O_2_). Superoxide anion radical initiates a cascade of reactions leading to the production of other ROS. The major sources for superoxide include nicotinamide adenine dinucleotide phosphate (NADPH) oxidases, mitochondrial respiration chain, xanthine oxidase, cyclooxygenases, and lipoxygenases [22]. Our body is equipped with a variety of antioxidants that serve to counterbalance the effect of ROS. These antioxidant defense systems consist mainly of superoxide dismutase (SOD), glutathione peroxidase (GPx), catalase, glutathione reductase (GR), and non-enzymatic antioxidants like vitamins and glutathione (GSH) [23]. However, in compromised pregnancy, the antioxidant system can be overwhelmed. Consequently, accumulating oxidative damage such as DNA damage, misfolded proteins, lipid peroxidation, and mitochondrial dysfunction, can trigger programmed processes leading to fetal programming and adverse offspring outcomes [14,24].

**Figure 1:**
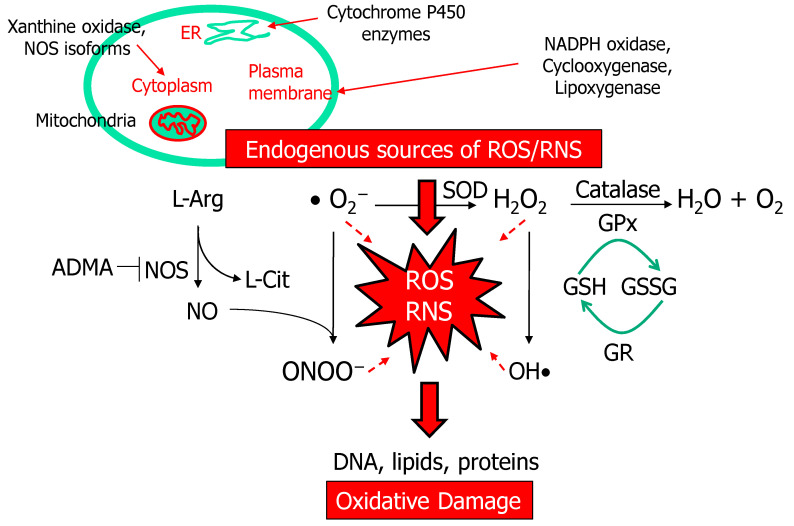
Schema outlining the reactive oxygen species (ROS)- and reactive nitrogen species (RNS)-generating pathways and antioxidant defense systems. Both the ROS and RNS are derived from endogenous sources like mitochondria, endoplasmic reticulum (ER), cytoplasm, and plasma membrane etc. Many enzymes produce superoxide radical (O_2_^−^) intracellularly, including NADPH oxidase, mitochondrial respiration chain, xanthine oxidase, cytochrome P450 enzymes, cyclooxygenase, and lipoxygenase. Nitric oxide synthase (NOS) catalyzes l-arginine (l-Arg) to generate nitric oxide (NO) and l-citrulline (l-Cit). However, uncoupled NOS produces superoxide, which scavenges NO leading to the peroxynitrite (ONOO^−^) formation. On the contrary, excessive ROS can be offset by the action of antioxidant enzymes. The components of antioxidant defense are superoxide dismutase (SOD), glutathione peroxidase (GPx), catalase, and glutathione reductase (GR). Reduced glutathione (GSH), a radical scavenger, is converted into oxidized glutathione (GSSG) through GPx and converted back to GSH by GR. Oxidative stress is a condition where ROS overwhelms the antioxidant system, which leads to cellular injury in the form of damaged DNA, lipids and proteins. RNS are produced starting with the reaction of superoxide and NO to form peroxynitrite.

NO is produced by the conversion from l-arginine into l-citrulline by NO synthase (NOS) that requires the cofactors. There are three different isozymes of NOS: endothelial NOS (eNOS), inducible NOS (iNOS) and neuronal NOS (nNOS). In the kidney constitutive NOS isoforms, mainly nNOS and eNOS, are expressed in physiological conditions, but iNOS is more likely to express under a pathological state [25]. In certain conditions like inhibition by NOS inhibitor asymmetric dimethylarginine (ADMA) [26], NOS possesses the unique ability to be uncoupled to produce superoxide, which scavenges NO leading to the peroxynitrite (ONOO^−^) formation. Peroxynitrite is the most detrimental RNS with marked cytotoxic effects. Accordingly, reduced NO bioavailability as a result of NOS uncoupling has been linked to kidney disease progression [17,25]. The ROS- and RNS-generating pathways and major defensive antioxidant systems are exemplified in Figure 1.

### 2.2. Redox State During Pregnancy

At various stages of pregnancy, fetal consumption differs. Fetal oxygen requirement is low during the first trimester. Low physiological oxygen concentrations are the naturally preferred microenvironment essential for organogenesis and differentiation. During the second and third trimesters, increased oxygen levels are needed for rapid gain of fetal weight and formation of the fetal–placental circulation [27]. Many studies have demonstrated elevated markers of oxidative stress in various complications of pregnancy, such as gestational diabetes, preterm birth, preeclampsia, and intrauterine growth retardation (IUGR) [13,14]. Accordingly, ROS acts like a double-edged sword in pregnancy, as an appropriate level of ROS is essential for normal fetal growth and development while produced at high level adversely affect the developing fetus [13]. Likewise, the double-faced role of NO in pregnancy is determined by its concentration. A moderate physiological level of NO is essential to maintain a healthy pregnancy [15]. Conversely, a high level of NO can react rapidly with superoxide to form peroxynitrite (ONOO^−^), a highly detrimental RNS with marked injurious effects.

### 2.3. Oxidative Stress in Fetal Programming

Critical role of oxidative stress implicated in fetal programming is supported by several lines of evidence. First, several obstetric and fetal complications are associated with oxidative stress, such as gestational diabetes, hypertension, preeclampsia, and preterm birth [13,14,28]. Second are experimental studies of animal models of renal programming. As we reviewed elsewhere [3], oxidative stress impacts renal programming in offspring born of dams exposed to diverse early-life insults, such as caloric restriction [29,30], low-protein diet [31], maternal diabetes [32], preeclampsia [33,34], prenatal dexamethasone exposure [35], prenatal dexamethasone and postnatal high-fat diet [36], and maternal smoking [37]. Third are observations that NO-ROS imbalance can precede kidney disease and hypertension, an early sign of CKD [16,17,18,38]. Renal NO deficiency develops early in young spontaneously hypertensive rats (SHRs) at four weeks of age, even before the onset of hypertension [39]. Similarly, studies in young SHR show increased NADPH oxidase expression and lipid peroxidation, while blood pressure does not rise yet [40]. Conversely, recent animal studies have indicated that early restoration of the NO-ROS balance could be reprogramming strategies to prevent the developmental origins of hypertension and kidney disease [18]. Fourth, ADMA per se can cause reduction of NO and production of ROS [27]. As in our previous report, ADMA-treated embryonic kidneys (metanephroi) results in dose-dependent decreases of nephron number [32]. Increased plasma ADMA levels are associated with adverse pregnancy and fetal outcomes such as preeclampsia [41], gestational diabetes [42], hypertension [43], IUGR [44], and prematurity [45].

## 3. Developmental Origins of Kidney Disease

### 3.1. Kidney Development

Nephron, functional unit of the kidney, comprises of Bowman’s capsule, glomerulus, and a tubule. In humans, there are about 1,000,000 nephrons in each kidney, with a 10-fold difference among individuals [46]. Nephrogenesis begins at week three and completes at around 36 weeks of gestation in humans [47]. In the rat, nephrogenesis continues after birth and ceases at one to two weeks postnatally [48]. In mammals, the metanephric kidney develops through interactions between the metanephric mesenchyme and ureteric bud (UB) [49]. The major developmental events include UB formation, UB branching morphogenesis, formation of bladder and kidney, kidney tubule branching, and nephrogenesis [49]. Impaired branching morphogenesis and nephrogenesis can cause low nephron endowment and a spectrum of defects in the kidney and urinary tract, namely congenital anomalies of the kidney and urinary tract (CAKUT) [50]. The developing kidneys are vulnerable to environmental risk factors that impair development throughout gestation: a malformed kidney is a severe defect happening during early pregnancy, whereas defects that occur later are generally less severe [50].

CKD is probably the result of interactions among multiple hits [51], a low nephron number likely creates a first-hit to the kidney, which increases the vulnerability of remaining glomeruli to develop CKD when facing suboptimal environment in later life. Epidemiological studies examining the risk factors for CKD indicate that maternal gestational diabetes, maternal obesity, low birth weight (LBW), and premature birth are associated with CKD [52]. Importantly, LBW and prematurity are related to low nephron number [52,53]. Additionally, a case-control study in Taiwan consisting of 1.6 million infants reported that risk factors for CAKUT consist of LBW, prematurity, maternal thalassemia, male, oligohydramnios or polyhydramnios, gestational diabetes, and first parity [54]. The role of low nephron number in renal programming is gaining attention is due to it can cause glomerular hyperfiltration, compensatory glomerular hypertrophy, and accordingly initiating a vicious cycle of further reduction of nephron [55]. However, there is no accurate and safe method for determining nephron in clinical practice so far.

### 3.2. Oxidative Stress-Related Renal Programming in Animal Models

So far, little is known about the impact of early-life oxidative stress on the development of kidney disease in humans. Animal models enables researchers to invoke various early-life insults at specific windows of development to determine their influence on programming processes and offspring outcome. Animal models particularly have provided more direct insight into the association between oxidative stress and kidney disease of developmental origins.

The present review is simply restricted to environmental insults starting in pregnancy and lactation period, with a focus on oxidative stress-related renal programming. Table 1 shows a range of adverse conditions during pregnancy and lactation may affect kidney development, resulting in morphological changes, functional adaption, and adverse renal outcomes in adulthood [29,30,31,32,33,34,35,56,57,58,59,60,61,62,63,64,65,66,67,68,69,70,71].

As shown in Table 1, maternal malnutrition is the most common factor to induce kidney disease of developmental origins. The range of nutritional insults can be grouped into different models that aim to restrict calorie intake [29,30], restrict protein intake [31], and increase consumption of diet with high level of fructose [56,58], fat [63], or methyl-donor [59]. Another factor interfering with renal programming is exposure to environmental toxins, such as 2,3,7,8-tetrachlorodibenzo-p-dioxin (TCDD) [62], bisphenol A [63], di-n-butyl phthalate [67], and smoking [69]. Additionally, maternal illness, such as diabetes [32], preeclampsia [33,34], CKD [60], reduced uterine perfusion [64], hypertension [66], and inflammation [67] have all been reported to affect kidney development and contribute to developmental programming of kidney disease. Additionally, renal programming can be triggered by medication like glucocorticoid [35,57,61,71].

Rats are the most commonly used animals. Kidney development in rats, unlike humans, continues up to postnatal week 1–2. Hence, suboptimal environmental conditions during pregnancy and lactation can impair nephrogenesis, consequently resulting in adult kidney disease. Given that rats reach sexual maturity at six weeks of age, and that one rat month is comparable to three human years in adulthood [72], Table 1 listing the timing of developing renal phenotypes allow calculations to refer to human ages.

### 3.3. Nephron Number and Oxidative Stress

Prior animal studies demonstrated that there are vulnerable periods during the development of kidney for developing a reduced nephron endowment [3]. Maternal insults need solely last for a brief period kidney development, as short as one to two days, to cause a long-lasting reduction of nephron number [73,74]. In a prenatal dexamethasone exposure model [74], treatments only reduced nephron number for periods of 48 h over days 13–14 and 17–18. As we reviewed elsewhere [3], a variety of maternal insults have been linked to low nephron endowment. However, the interrelationship between oxidative stress and reduced nephron number solely reported in the caloric restriction model [29], streptozotocin-induced diabetes [32], and maternal smoking [70]. Our previous study reported that ureteric bud branching morphogenesis was inhibited by ADMA, a ROS inducer and endogenous NOS inhibitor, consequently leading to decreases of nephron number [32]. Collectively, these studies support the impact of oxidative stress on low nephron endowment. However, reduced nephron number per se does not appear to mediate all programmed processes underlying developmental origins of kidney disease, as nephron number can be unaltered [66], or even increased in response to renal programming [75]. In addition to reduced nephron number, glomerular hypertrophy [29,30,60,68] and tubulointerstitial injury [29,30,32,66,68] are major morphological deficits associated with renal programming (Table 1).

On the other hand, the most common phenotype of renal programming being studied is hypertension [29,30,31,32,33,34,35,56,58,59,60,61,62,63,64,65,67,71]. Albuminuria has been demonstrated in offspring mice born of dams with high-fat intake [69] or maternal smoking exposure [70]. Additionally, renal function was not affected in most models of renal programming [29,30,32,59,60], except one report demonstrated blood creatinine level was elevated in 12-week-old offspring born to dams received dexamethasone administration during lactation [57]. Our review implicates that the renal programming does not rely on one particular factor exposure and it show a variety of phenotypes. Whether there are common mechanisms contributing to various renal phenotypes deserves further clarification.

### 3.4. Reported Mechanisms of Oxidative Stress in Renal Programming

Several mechanisms of oxidative stress have been reported involving the pathogenesis of renal programming, including increased production of ROS [64,66,67,69,70], decreased capabilities of endogenous antioxidant systems [31,57], impaired ADMA-NO pathway [32,33,35,60,63], and increased oxidative damage [29,30,31,34,56,57,58,59,61,62,63,64,65,67,68]. Two of the most well studied markers of lipid peroxidation are F_2_-isoprostanes and malondialdehyde (MDA) [76]. As shown in Table 1, renal programming induced by maternal low protein diet [31], maternal l-NAME administration [34], maternal dexamethasone administration [57], reduced uterine perfusion [64], and prenatal lipopolysaccharide (LPS) exposure [67] is relevant to oxidative damage of lipid peroxidation. Additionally, 8-hydroxydeoxyguanosine (8-OHdG), an oxidized nucleoside of DNA, is the most frequently detected and studied oxidative stress induced DNA lesion [76]. Maternal high-fructose diet [56,58], maternal methyl-deficient or -donor diet [59], prenatal dexamethasone exposure [61], prenatal dexamethasone plus TCDD exposure [62], combined bisphenol A and high-fat diet exposure [63], and high-fat diet [65,69] have given rise to renal programming and programmed kidney disease in the presence of increased renal 8-OHdG expression. As we reviewed elsewhere [77], early-life ADMA-related NO-ROS imbalance may cause long-lasting functional and structural changes in later life in the kidney. This is the case for ADMA that is a key risk factor related to renal programming in a variety of programming models, including maternal caloric restriction [29,30], gestational diabetes [32], maternal CKD [60], prenatal dexamethasone plus TCDD exposure [62], and prenatal bisphenol A exposure plus high-fat diet [63].

## 4. Targeting Oxidative Stress by Antioxidants as a Reprogramming Strategy

### 4.1. Antioxidants

Antioxidants can be classified as either enzymatic or non-enzymatic based on their activity [78]. The human body is equipped with enzymatic and nonenzymatic antioxidants to catalyze the ROS/RNS and protect the cells against oxidative damage. There are two groups of non-enzymatic antioxidants: natural and synthetic antioxidants [79]. Examples of natural nonenzymatic antioxidants are vitamin E, A, C, flavonoids, glutathione, carotenoids, and polyphenols [78]. Natural antioxidants come mainly from plants, such as vegetables, nuts, fruits, and seeds. As they can be easily used for dietary interventions, the consumption of foods with high antioxidant potential is quite important. As reviewed elsewhere [19,20,80,81], several commonly used antioxidants like vitamins C, E, l-arginine, coenzyme Q10 (CoQ10), and *N*-acetylcysteine (NAC) have been taken to reduce oxidative stress in human trials for CKD. Nevertheless, to date specific antioxidants are not yet recommended by therapeutic guideline for CKD therapy [81].

### 4.2. Antioxidant Therapy as a Reprogramming Strategy

**Figure 2:**
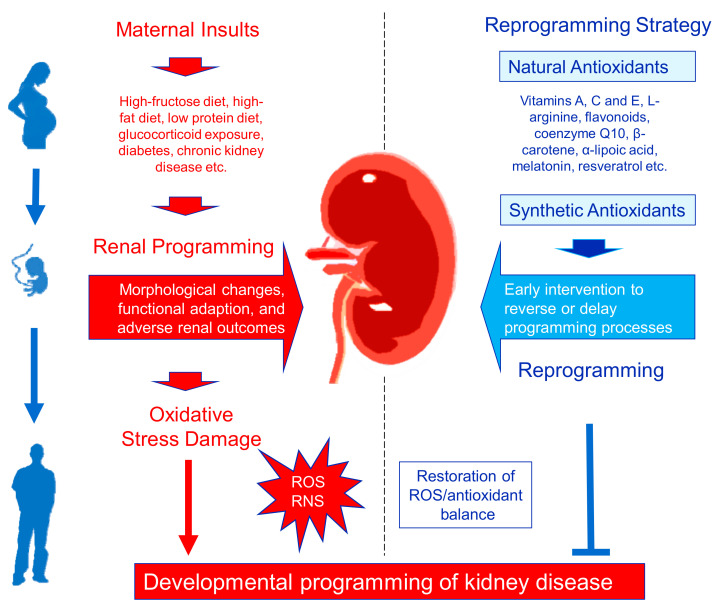
Schema outlining the programming versus reprogramming effects on the kidney. Multiple maternal insults can induce renal programming and oxidative stress, consequently leading to kidney disease in adulthood. Oxidative stress is an imbalance between excessive reactive oxygen species (ROS)/reactive nitrogen species (RNS) and impaired antioxidant defenses, in favor of the first. Conversely, perinatal use of natural and synthetic antioxidants may reverse or delay programmed processes to prevent the development of kidney disease by so-called reprogramming. The beneficial effects of reprograming interventions on renal programming are related to the restoration of ROS/antioxidant balance.

The ultimate goal of all research on adult disease of developmental origins is to develop preventive interventions to delay or reverse programmed processes by so-called reprograming [10]. Given that oxidative stress plays a decisive role in programmed kidney disease, it is a reasonable assumption that antioxidant therapy would be reprogramming strategies to optimize offspring’s renal outcomes. A summary of the link between various maternal insults implicated in renal programming and reprogramming by antioxidant therapy to prevent developmental programming of kidney disease is depicted in Figure 2.

Nevertheless, it is largely unknown whether supplementing with antioxidants in pregnancy and lactation can be beneficial on developmental origins of kidney disease in humans. We confined this review to antioxidants implemented mainly during gestation and lactation which are critical periods for kidney development. As listed in Table 2, we summarize current knowledge on perinatal use of antioxidants used as a reprogramming strategy to protect offspring against renal programming in various animal models [29,30,31,33,34,35,36,59,60,62,63,67,70,82,83,84,85,86,87,88,89,90,91,92,93,94,95,96,97]. As the DOHaD research is a flourishing field, this list is likely to grow rapidly.

### 4.3. Vitamins

Vitamins C and E are the most commonly used antioxidants. Vitamin C (ascorbic acid), a six-carbon ketolactone, possesses an ability to quench ROS. Vitamin E (α-tocopherol) functions as an essential lipid soluble antioxidant that inhibits lipoxygenase, NADPH oxidase, and cyclooxygenase [98]. Vitamin E is often delivered with vitamin C to boost its antioxidant efficacy. Vitamin C, E, alone or combined other antioxidants have been used in pregnancy and lactation for kidney disease of developmental origin [67,82,83,84,85]. Gestational use of Vitamin C or E alone protected adult offspring against maternal LPS exposure induced hypertension [67,82]. Additionally, perinatal supplementation of vitamins C, E, l-arginine, and l-taurine can prevent hypertension in SHR [83,84], and kidney injury in Fawn hooded hypertensive rat (FHH) [85]. However, the above mentioned findings in animal models have not been explored yet in relation to the possible benefits in clinical translation.

### 4.4. Amino Acids

Several amino acids have been studied as a dietary antioxidant supplement to alleviate oxidative stress [29,32,35,60,83,84,85,86,87]. l-taurine has been used with other antioxidants to prevent the development of hypertension in SHRs and FHH rats; the two most widely studied genetic hypertensive rat models [83,84,85]. Taurine, a non-protein sulfur amino acid, is the most abundant intracellular amino acid [99]. Perinatal l-taurine supplements combined with other antioxidants showed protective effects against proteinuria and glomerulosclerosis in SHRs [85], which ties well with previous studies demonstrating that l-taurine therapy acts as a renoprotective agent in established kidney disease [100,101].

As NO deficiency is one of the pathogenetic mechanisms underlying kidney disease [16,17], two amino acids, l-arginine (the substrate of NOS) and l-citrulline (a precursor to l-arginine) in the NO pathway, have been studied to ameliorate kidney disease [102,103,104]. So far, the benefits of l-arginine from human trials are still inconclusive [105]. Although perinatal use of l-arginine combined with other antioxidants have shown benefits on renal programming [83,84,85], whether l-arginine supplementation alone in pregnancy will accompany with these beneficial effects awaits further clarification. Unlike l-arginine, l-citrulline can bypass hepatic metabolism and can be converted to arginine in the kidney [103]. Following oral citrulline supplementation, blood l-arginine levels reach their peak after 1–2 h [103]; Oral l-citrulline supplementation has been applied to increase l-arginine production and to raise NO levels [104]. So far, experimental evidence suggests that there are beneficial effects of perinatal l-citrulline supplementation on renal programming in several animal models, including maternal caloric restriction [29], maternal streptozotocin (STZ)-induced diabetes [32], and prenatal dexamethasone exposure [35]. In SHR, perinatal use of l-citrulline can restore NO bioavailability and prevent the transition of prehypertension to hypertension [86]. Additionally, l-tryptophan and branched-chain amino acids (BCAAs) have also been assessed as reprogramming interventions in models of maternal CKD [60] and maternal caloric restriction [87], respectively.

Although there has been a focus on antioxidant effects of amino acids and increasingly investigated for their reprogramming potentials on renal programming, there remains an unmet need for the accurate dietary recommendations of amino acid requirements for women during pregnant and lactation.

### 4.5. Melatonin

Melatonin, an endogenous indoleamine synthesized from l-tryptophan, is mainly secreted by the pineal gland [106]. Although melatonin is broadly present in many foods from animals and plants, practically all melatonin supplements that are marketed are made from synthetic melatonin [107]. Melatonin possesses pleiotropically biological functions, including fetal development, regulation of circadian rhythm, anti-inflammation, epigenetic regulation, and antioxidant [106,108,109,110]. As a potent antioxidant, melatonin and its metabolites can scavenge ROS/RNS and upregulate antioxidant enzymes [110]. Melatonin has demonstrated benefits in some established kidney diseases, as review previously [111,112]. Additionally, perinatal use of melatonin has been considered as a reprogramming strategy to prevent certain adult diseases in diverse models of developmental programming [113,114].

Table 2 shows perinatal use of melatonin benefits on hypertension in several models of renal programming, such as maternal caloric restriction [30], maternal l-NAME exposure [34], maternal methyl-donor diet [59], maternal constant light exposure [88], high-fructose diet [89], high-fructose diet plus post-weaning high-salt diet [90], prenatal dexamethasone exposure [91], and prenatal dexamethasone exposure plus post-weaning high-fat diet [92]. The beneficial role of melatonin therapy is related to its antioxidant property, including diminished F_2_-isoprostane level [34], decreased ADMA concentrations [30,90], reduced 8-OHdG expression [30,59], and increased NO bioavailability [30,89,90]. Altogether, these findings make melatonin a potential reprogramming intervention to prevent kidney disease of developmental origin.

### 4.6. Resveratrol

Another natural antioxidant has been studied as a reprogramming intervention is resveratrol [115]. Resveratrol, a natural polyphenol, has been widely used as a nutritional supplement [116]. Its antioxidant properties include inhibiting NADPH oxidase, reducing ROS production, increasing glutathione level, and increasing the expression of various antioxidant enzymes [117]. Although resveratrol has shown nephroprotective effects in experimental kidney diseases [118], only limited number of studies exist examining its reprogramming effects in animal models of renal programming [58,62,63,94]. In a maternal TCDD and dexamethasone exposure model, perinatal resveratrol therapy benefits on renal programming coincided with a decrease of ADMA production, reduction of renal 8-OHdG expression, and an increase of NO bioavailability [62]. Likewise, resveratrol therapy restored the balance between ROS and NO and protected adult offspring against hypertension induced by maternal exposure to combined BPA and high-fat diet [63], high-fructose diet [94], and l-NAME plus postnatal high-fat diet [95].

### 4.7. Synthetic Antioxidants

Aside from natural antioxidants, some synthetic antioxidants have been applied in animal models of renal programming to improve offspring outcomes. Lazaroid, an inhibitor of lipid peroxidation, is a powerful scavengers of ROS [119]. One report demonstrated that maternal lazaroid therapy prevented hypertension programmed by maternal low protein diet in adult male rat offspring [31]. Next, MitoQ is a synthetic antioxidant, analogous to the natural antioxidant CoQ10. CoQ10 can attenuate oxidative stress by reduction of superoxide production, prevention of lipid peroxidation, and interactions with α-tocopherol [120]. There is general lack of studies investigating perinatal CoQ10 supplementation for the prevention of kidney disease of developmental origins. Only one study reported that perinatal use of MitoQ protected adult offspring against hypertension, kidney injury, and reduced nephron number in a maternal smoking mouse model [70]. Dimethyl fumarate (DMF) is a nuclear factor erythroid-derived 2-related factor 2 (Nrf2) activator [121]. Nrf2 is a well-known redox-sensing transcription factor which controls numerous genes that are involved in the management of oxidative stress [122]. In a maternal dexamethasone exposure and postnatal high-fat diet model, maternal DMF treatment protected adult progeny against hypertension coincided with reducing ADMA concentration, increasing NO bioactivity, and reducing renal 8-OHdG expression [96]. Moreover, tempol is a redox-cycling nitroxide that promotes the metabolism of ROS and improves NO bioavailability. Although tempol has been studied extensively in animal models of oxidative stress [123], only one study reported that perinatal use of tempol was capable of protecting adult SHRs against hypertension and proteinuria [96].

### 4.8. N-Acetylcysteine

*N*-acetylcysteine (NAC) is a thiol-containing synthetic compound used in the treatment of oxidative stress-related disorder [124]. NAC is a precursor to glutathione and a stable l-cysteine analogue for H_2_S synthesis [125]. The implication of perinatal NAC therapy in renal programming is evidenced by the protection against hypertension in adult offspring born of dams exposed to suramin [33], l-NAME [34], and dexamethasone combined with postnatal high-fat diet [36]. In the prenatal dexamethasone and postnatal high-fat diet model, the beneficial effects of NAC against offspring hypertension were associated with increased H_2_S-generating enzyme expression, increased plasma glutathione level, and reduction of renal 8-OHdG expression [36]. In another study, perinatal use of NAC also protected SHR offspring against hypertension via increased expression and activity of renal H_2_S-generating enzymes and activity [97]. Furthermore, NAC therapy in pregnancy and lactation prevented hypertension programmed by maternal suramin administration, which coincided with increased GSH and restoration of NO [33].

## 5. Concluding Remarks and Perspectives

The above-mentioned evidence supports that maintaining a physiological oxidative-antioxidative balance is advised to prevent developmental origins of kidney disease. However, there are many aspects still unsolved. At face value, it would be logical to consider early-life antioxidant interventions in potential reprogramming therapies for kidney disease of developmental origins. However, it is noteworthy that excessive antioxidant supplement may shift oxidative stress to an opposite state of “antioxidant stress” [126]. That is, perinatal antioxidant supplement would only apply in the case of deficits, but not as a usual intake.

Another important aspect is that the use of antioxidant targeted to specific pathways that are impaired in renal programming proves beneficial, while several antioxidants may be needed as a multi-drug therapy to target oxidant modifying pathways during kidney development. At a deeper level, there is still a long road ahead for clinical translation to determine the “right” antioxidant with the “right” dose for the “right” person to reprogram renal programming-related diseases. Therefore, future work in large prospective trials is needed to better assess the relationship between oxidative stress and kidney disease of developmental origins.

Renal programming, apart from oxidative stress, has been linked to other common molecular mechanisms [2,3,6,8]. On the other hand, several beneficial mechanisms have been reported related to the reprogramming effects of perinatal antioxidant therapy on developmental programming [21], such as activation of nutrient-sensing signals, rebalancing of the renin-angiotensin system (RAS), and reshaping gut microbiota. Therefore, oxidative stress seems not alone lead to renal programming. What is missing from the literature is a greater understanding of how oxidative stress interrelates with these common mechanisms to affect renal programming, and whether interventions targeting other mechanisms (e.g., RAS blocker or probiotics) in pregnancy may also reduce oxidative stress to prevent offspring against kidney disease of developmental origins.

In conclusion, oxidative stress is a significantly pathogenetic link for developmental origins of kidney disease. Further research are needed to get insight into the mechanisms underlying renal programming and specific antioxidant as reprogramming therapies to reduce the global burden of kidney disease.

## Figures and Tables

**Table 1:** Summary of oxidative stress-related renal programming following maternal insults in animal models.

Animal Models	Species/Gender	Age at Evaluation	Mechanisms of Oxidative Stress	Morphological Changes	Renal Phenotype	Ref.
Maternal caloric restriction diet, 50%	SD rat/M	12 weeks	↑ ADMA, ↓ NO, ↑ Renal 8-OHdG expression	↓ NN, glomerular hypertrophy, ↑ tubulointerstitial injury	↔ GFR, hypertension	[29,30]
Maternal low protein diet, 9%	Wistar rat/M	12 weeks	↑ F_2_-isoprostane, ↓ glutathione		Hypertension	[31]
Streptozotocin-induced diabetes	SD rat/M	12 weeks	↑ ADMA, ↓ NO	↓ NN, ↑ tuburo-interstitial injury	↔ GFR, hypertension	[32]
Maternal suramin administration	SD rat/M	12 weeks	↑ ADMA, ↓ NO		Hypertension	[33]
Maternal l-NAME administration	SD rat/M	12 weeks	↑ Renal F_2_-isoprostane level		Hypertension	[34]
Maternal high-fructose diet, 60%	SD rat/M	12 weeks	↑ Renal 8-OHdG expression, ↓ NO		Hypertension	[56]
Dexamethasone administration in lactation	Wistar rat/M and F	12 weeks	↑ Renal MDA level, ↓ SOD and catalase activity	↑ Tubular necrosis	↑ Cr level	[57]
Maternal plus post-weaning high-fructose diet, 60%	SD rat/M	12 weeks	↑ Renal 8-OHdG expression		Hypertension	[58]
Maternal methyl-deficient diet	SD rat/M	12 weeks	↑ Renal 8-OHdG expression		↔ Cr level, hypertension	[59]
Maternal high methyl-donor diet	SD rat/M	12 weeks	↑ Renal 8-OHdG expression		↔ Cr level, hypertension	[59]
Maternal adenine-induced CKD	SD rat/M	12 weeks	↑ ADMA, ↓ NO	Renal hypertrophy	↔ Cr level, hypertension	[60]
Prenatal dexamethasone at gestational day 15 and 16.	SD rat/M	16 weeks	↓ Renal NO		Hypertension	[35]
Prenatal dexamethasone exposure plus postnatal high-fat intake	SD rat/M	16 weeks	↑ Renal 8-OHdG expression, ↓ NO		Hypertension	[61]
Prenatal dexamethasone plus TCDD exposure	SD rat/M	16 weeks	↑ Renal 8-OHdG expression, ↑ ADMA		Hypertension	[62]
Prenatal bisphenol A exposure plus high-fat diet	SD rat/M	16 weeks	↑ Renal 8-OHdG expression, ↑ ADMA, ↓ NO		Hypertension	[63]
Reduced uterine perfusion	SD rat/M	16 weeks	↑ Urinary F_2_-isoprostane level and renal NADPH-oxidase dependent superoxide		Hypertension	[64]
Maternal plus post-weaning high-fat diet, 58%	SD rat/M	16 weeks	↑ Renal 8-OHdG expression		Hypertension	[65]
Maternal angiotensin II administration	Wistar rat/M	18 weeks	↑ Renal ROS	↔ NN, ↑ tuburo-interstitial injury		[66]
Prenatal LPS Exposure	Wistar rat/M	28 weeks	↑ Renal MDA level		Hypertension	[67]
Maternal di-n-butyl phthalate exposure	SD rat/M and F	18 months	↑ Renal ROS	Renal dysplasia, ↑ tuburo-interstitial injury		[68]
Maternal high-fat diet	C57BL/6 mice/M	9 weeks	↑ Renal 8-OHdG expression	Renal hypertrophy	Albuminuria	[69]
Maternal smoking exposure	Balb/c mice/M	13 weeks	↑ Renal ROS	↓ NN	Albuminuria	[70]
Prenatal betamethasone exposure at gestational day 80 and 81	Sheep/M and F	18 months	↑ ROS, ↓ NO		Hypertension	[71]

Studies tabulated according to species and age at evaluation. ADMA = asymmetric dimethylarginine; CKD = chronic kidney disease; Cr = creatinine; LPS = lipopolysaccharide. SD = Sprague Dawley; M = male; F = female; GFR = glomerular filtration rate; l-NAME = l-N^G^-Nitro arginine methyl ester; MDA = malondialdehyde; SOD = superoxide dismutase; NN = nephron number; TCDD = 2,3,7,8-tetrachlorodibenzo-p-dioxin; 8-OHdG = 8-hydroxy-2′–deoxyguanosine; ↑= increased; ↓= decreased; ↔ = unaltered.

**Table 2:** Summary of antioxidants used as reprogramming interventions in animal models of renal programming.

Antioxidant Intervention	Animal Models	Species/Gender	Age at Evaluation (Week)	Reprogramming Effects	Ref.
Natural antioxidants					
Vitamin C 350 mg/kg/day i.p. daily at gestational day 8 to 14	Prenatal LPS Exposure	SD rat/M	12	↓ BP	[82]
α-tocopherol 350 mg/kg/day via gavage at gestational day 13 to 20	Prenatal LPS Exposure	Wistar rat/M	28	↓ BP	[67]
l-arginine, l-taurine, Vitamins C and E 2 weeks before until 8 weeks after birth	Genetic hypertension	SHR/M and F	9	↓ BP	[83]
l-arginine, l-taurine, Vitamins C and E 2 weeks before until 8 weeks after birth	Genetic hypertension	SHR/M and F	50	↓ BP, ↓ proteinuria	[84]
l-arginine, l-taurine, Vitamins C and E 2 weeks before until 4 weeks after birth	Genetic hypertension	FHH rat/M and F	36	↓ BP, ↓ proteinuria, ↓ glomerulosclerosis	[85]
0.25% l-citrulline in drinking water in pregnancy and lactation	Maternal caloric restriction diet, 50%	SD rat/M	12	↓ kidney injury, ↑ nephron number	[29]
0.25% l-citrulline in drinking water in pregnancy and lactation	Maternal streptozotocin -induced diabetes	SD rat/M	12	↓ BP, ↓ kidney injury	[32]
0.25% l-citrulline in drinking water in pregnancy and lactation	Prenatal dexamethasone exposure	SD rat/M	12	↓ BP	[35]
0.25% l-citrulline in drinking water 2 weeks before until 6 weeks after birth	Genetic hypertension	SHR/M and F	50	↓ BP	[86]
l-tryptophan 200 mg/kg BW/day via oral gavage in pregnancy	Maternal adenosine-induced CKD	SD rat/M	12	↓ BP, ↓ Cr level	[60]
BCAA-supplemented diet in pregnancy	Maternal caloric Restriction, 70%	SD rat/M	16	↓ BP	[87]
0.01% melatonin in drinking water in pregnancy and lactation	Maternal caloric restriction	SD rat/M	12	↓ BP	[30]
0.01% melatonin in drinking water in pregnancy and lactation	Maternal l-NAME exposure	SD rat/M	12	↓ BP	[34]
0.01% melatonin in drinking water in pregnancy and lactation	Maternal methyl-donor diet	SD rat/M	12	↓ BP	[59]
0.01% melatonin in drinking water in pregnancy and lactation	Maternal constant light exposure	SD rat/M	12	↓ BP	[88]
0.01% melatonin in drinking water in pregnancy and lactation	Maternal high-fructose diet, 60%	SD rat/M	12	↓ BP	[89]
0.01% melatonin in drinking water in pregnancy and lactation	Maternal high-fructose diet plus post-weaning high-salt diet	SD rat/M	12	↓ BP	[90]
0.01% melatonin in drinking water in pregnancy and lactation	Prenatal dexamethasone exposure	SD rat/M	16	↓ BP	[91]
0.01% melatonin in drinking water in pregnancy and lactation	Prenatal dexamethasone exposure plus post-weaning high-fat diet	SD rat/M	16	↓ BP	[92]
Melatonin 10 mg/kg/day in drinking water in pregnancy	Genetic hypertension model	SHR/M	16	↓ BP	[93]
0.05% resveratrol in drinking water in pregnancy and lactation	Maternal TCDD and dexamethasone exposures	SD rat/M	16	↓ BP	[62]
50 mg/L resveratrol in drinking water in pregnancy and lactation	Maternal bisphenol A exposure and high-fat diet	SD rat/M	16	↓ BP	[63]
50 mg/L resveratrol in drinking water in pregnancy and lactation	Maternal plus post-weaning high-fructose diet	SD rat/M	12	↓ BP	[58]
50 mg/L resveratrol in drinking water in pregnancy and lactation	Maternal l-NAME plus postnatal high-fat diet	SD rat/M	16	↓ BP	[94]
Synthetic antioxidants					
Lazaroid 10 mg/kg/day via gavage in pregnancy	Maternal low protein diet, 9%	Wistar rat/M	12	↓ BP	[31]
MitoQ 500 µM in drinking water in pregnancy and lactation	Maternal smoking exposure	Balb/c mice/M	13	↓ BP, ↓ kidney injury, ↑ nephron number	[70]
Dimethyl fumarate 50 mg/kg/day via gavage in pregnancy	Prenatal dexamethasone and postnatal high-fat diet	SD rat/M	16	↓ BP	[95]
Tempol 172 mg/L in drinking water 2 weeks before until 8 weeks after birth	Genetic hypertension	SHR/Mand F	50	↓ BP, ↓ proteinuria	[96]
1% NAC in drinking water in pregnancy and lactation	Suramin administration	SD rat/M	12	↓ BP	[33]
1% NAC in drinking water in pregnancy and lactation	Maternal l-NAME exposure	SD rat/M	12	↓ BP	[34]
1% NAC in drinking water in pregnancy and lactation	Prenatal dexamethasone and postnatal high-fat diet	SD rat/M	12	↓ BP	[36]
1% NAC in drinking water in pregnancy and lactation	Genetic hypertension model	SHR rat/M	12	↓ BP	[97]

Studies tabulated according to types of antioxidant intervention, animal models and age at evaluation. BP = blood pressure. CKD = chronic kidney disease. Cr = creatinine. FHH = Fawn hooded hypertensive rat. L−NAME = N^G^−nitro−L−arginine−methyl ester. M = male. F = female. NAC = N-acetylcysteine. BCAA = branched-chain amino acid. LPS = lipopolysaccharide. SD = Sprague-Dawley rat. SHR = spontaneously hypertensive rat. TCDD = 2,3,7,8-tetrachlorodibenzo-p-dioxin; ↑= increased; ↓= decreased.

## Data Availability

Not applicable.
